# Leveraging Genetic Diversity for Abiotic Stress Tolerance

**DOI:** 10.3390/ijms27104258

**Published:** 2026-05-11

**Authors:** Andrés J. Cortés

**Affiliations:** Facultad de Ciencias Agrarias—Departamento de Ciencias Forestales, Universidad Nacional de Colombia—Sede Medellín, Medellín 050034, Colombia; ancortesv@unal.edu.co

Climate-change-driven abiotic stresses, once regarded as episodic constraints for major staple crops, are now main selective forces driving the agronomic and evolutionary trajectories of plants [1]. Increasing climatic variability has been manifested in the last decade as recurrent and extreme droughts [2], heatwaves [3], and cold fronts [4,5,6,7], which together are pushing crops and natural populations toward the limits of their plastic [8] and adaptive capacity [9]. Plant breeding and management efforts have buffered some of these challenges [10,11], yet they have often relied on relatively narrow genetic bases and a reductionist set of productive [12] and eco-physiological functional traits [13]. Since this standing variation is insufficient to cope with the rapid pace of environmental change [14,15], an urgent expansion in the repertoire of nature-based solutions is demanded. In particular, boosting abiotic stress tolerance would benefit by expanding the classical recurrent trait improvement cycle and germplasm mobilization approaches to encompass a high-throughput exploration and introgression of hidden allelic variation [16], as well as genetic forecasting of the adaptative [17] and conversation potentials [18,19,20,21]. For that, modern and more integrated molecular, ecological, computational, and trait discovery and screening approaches must be envisioned [22]. In order to help bridge this gap, this second edition of the Special Issue “Abiotic Stress Tolerance and Genetic Diversity in Plants” has assembled nine contributions, spanning a rich variety of scales, from gene networks to population-level studies. This compilation reinforces the fact that abiotic stress tolerance is a complex, multi-dimensional, polygenic, and environmentally dependent abstractable property [23], rather than a simple predefined phenotype encoded by few deterministic genes with major effects. While abiotic stresses impact plant productivity and survival under climate change, genetic and omic platforms are converging to reshape strategies for breeding more resilient crops and maintaining species ecosystem services.

## 1. Molecular and Transcriptomic Bases of Abiotic Stress Responses

Understanding how plants perceive, process, and respond to abiotic stress at the molecular level remains a central challenge for advancing climate-resilient agriculture and conservation strategies [24]. Rapid progress in transcriptomics, comparative genomics, and systems biology has enabled a shift from single-gene studies to family- and network-based assessments, revealing the complex regulatory architectures that underlie stress adaptation [9,25]. These approaches are capable of uncovering cryptic functional variation within wild relatives [26], locally adapted germplasm [27,28], and polyploid genomes [29]—sources of innovation that, until very recently, have been systematically deployed into breeding and conservation programs. As discussed below, gene family evolution, functional validation, and temporal expression dynamics help to determine how transcriptional regulation shapes plant responses to environmental pressures [30,31,32].

As a first case study that goes beyond primary gene pools, the contribution by Wong et al. [33] investigates cold tolerance response mechanisms by comparing cultivated alfalfa (*Medicago sativa*) with its wild relative *Medicago falcata*. The authors expose seedlings of both species to low-temperature treatment (4 °C) as well as to control conditions, and analyze leaf tissues at defined time points using RNA-seq, differential gene expression, and transcription factor enrichment analyses. The results show that *M. falcata* exhibits a broader transcriptional response with significantly more differentially expressed genes (DEGs) and enrichment in key regulatory families such as bHLH, ARR-B, and AP2/ERF [34,35,36,37,38,39]. The authors conclude that wild germplasm harbors a richer regulatory network for stress response than its cultivated counterparts, suggesting that domestication has eroded the diversity of stress-responsive pathways. It also illustrates how comparative transcriptomics uncover functionally relevant regulatory variation, reinforcing the importance of integrating wild genetic resources aimed at enhancing resilience.

Similarly, but this time looking into locally adapted germplasm, O’Rourke and Graham [40] provide a compelling example of how stress-resilient crop provenances can harbor novel adaptive mechanisms that are not predictable from model species. Focusing on soybean (*Glycine max*), the authors investigate the function of three iron deficiency response genes—FIT, HY5, and PYE—in the iron-deficit-tolerant Swedish line Fiskeby III (PI 438471), using virus-induced gene silencing combined with RNA-seq analyses under iron-sufficient and iron-deficient conditions. Strikingly, while silencing FIT and HY5 alters broader stress-responsive transcriptional networks, it does not compromise iron deficiency tolerance, demonstrating that this genotype relies on alternative, previously uncharacterized, genetic pathways to cope with iron scarcity. These findings challenge the widespread assumption that stress tolerance mechanisms are conserved across species, and highlight the limitations of extrapolating from model organisms such as *Arabidopsis thaliana*. Instead, the study encourages exploring crop-specific and locally adapted germplasm to uncover hidden sources of genetic diversity for stress resilience.

In a more temporal expression context, Khunsanit and collaborators [41] leverage time-series transcriptomics and network-based approaches to dissect the molecular basis of salinity tolerance in rice (*Oryza sativa*), focusing on the salt-tolerant Thai variety ‘Jao Khao’. Using 36 RNA-seq libraries sampled across six time points (0–48 h) under 160 mM NaCl, the authors construct a two-state weighted gene co-expression network that enables the identification of 1950 highly variable genes and 111 key hub genes associated with the salt stress response. These hub genes were enriched in pathways related to energy metabolism, photosynthetic light reactions, ATP synthesis, and transport processes, with early upregulation during the initial phases of salt stress, a pattern further validated by qRT-PCR for key genes such as RuBisCo and ATP synthase. The study demonstrates that rapid metabolic reprogramming, particularly the management of cellular energy balance, is transversal to stress adaptation. It also demonstrates how merging integrative transcriptomic and systems biology frameworks across the temporal continuum uncover functionally relevant candidate gene targets to harness abiotic stress responses in crops.

Finally, Zhao and collaborators [42] offer a comprehensive genome-wide characterization of the *FCS-like zinc finger (FLZ)* gene family, implied in stress responses as well as plant development and senescence, across three *Brassica* species, identifying 113 genes and revealing their expansion largely driven by whole-genome duplication events, well-studied events in *Brassica* genome evolution. Through integrative phylogenomic, collinearity, and expression analyses, combined with protein–protein interaction assays, the study demonstrates that a subset of these genes (i.e., seven out of 15 tested) shows significant responsiveness to abiotic stresses, while many FLZ proteins interact with central energy-sensing kinases (SnRK1), highlighting a conserved regulatory module linking stress signaling and metabolic control.

Overall, these studies offer a genome-wide functional exploration of stress-responsive genetic mechanisms in plants, highlighting how integrated approaches can uncover candidate genes linked to abiotic stress resilience [43,44]. The works combine high-throughput genomic screening and expression profiling under stress conditions, which enables authors to identify key gene families exhibiting differential regulation and potential roles in adaptive responses [45,46,47], while also revealing patterns of gene duplication and structural diversification responsible for functional innovation [48]. The studies move beyond descriptive genomics after linking molecular signatures and expression profiles to stress-related pathways, thereby contributing to a more mechanistic understanding of how plants perceive and respond to climate pressures, even at local scales [49,50]. They also exemplify how modern bioinformatics and molecular biology tools can reveal previously hidden layers of genetic diversity, both at the population and the polyploid genomic levels, offering promising targets for molecular breeding and the customization of climate-resilient populations [51]. Ultimately, these studies reinforce the value of integrating genomic, functional, and evolutionary analyses to unlock adaptive potential in plants [52].

## 2. Transitioning from Functional Bases to Targeted Gene Editing

Based on the molecular and transcriptomic understanding outlined above, the natural next step is to functionally validate and bioengineer the causal variants that are responsible for abiotic stress responses [53]. While genome-wide expression and comparative evolutionary analyses of major gene families have been key in identifying candidate genes and regulatory networks [54], they are often limited in terms of determining how specific alleles, isoforms, or regulatory elements translate into phenotypic resilience. Recent advances in functional genomics are beginning to bridge this gap by combining fine-scale molecular characterization with experimental validation across heterologous systems and genome editing platforms. These approaches not only disclose previously overlooked layers of variation—such as alternative splicing or regulatory sequence diversity—but also enable their direct manipulation for plant improvement. That said, the following studies illustrate how moving from gene discovery to mechanistic validation and targeted intervention enable a first approach to utilizing cryptic adaptive potential, accelerating the deployment of stress-resilient traits, and reinforcing the convergence of molecular biology, biotechnology, and breeding in the face of escalating abiotic challenges [55].

Illustrating the first scenario, Imran et al. [56] study functional variation in the sodium transporter *OsHKT1;1* for salt tolerance in rice (*Oryza sativa*). The authors test two splicing variants across three heterologous systems (i.e., plant cells, yeast, and *Xenopus oocytes*) using subcellular localization, ion flux measurements, and gene expression analyses. The team demonstrate that splicing variants differ in ion selectivity and transport efficiency, offering a cryptic source of variation for crop improvement. After all, not only gene presence, but also alternative splicing contributes to plasticity, expanding the functional repertoire of key transporters involved in ion homeostasis.

Meanwhile, at the frontiers of gene editing, Fan and collaborators [57] reviewed CRISPR/Cas-based genome editing [58,59] in alfalfa (*Medicago sativa*), a key forage crop with historically underexploited genomic resources. The work summarizes how merging conventional breeding with emerging genomic, transcriptomic, and functional studies enable identifying candidate genes and pathways associated with traits such as nutrient use efficiency and freezing tolerance, among other abiotic stress responses. The synthesis prospects multiplex gene editing, regulatory region targeting, and trait engineering strategies to boost these candidate targets. The authors claim that while genome editing enables precise manipulation of stress-related genes, challenges remain in transformation efficiency and the complexity of polygenic traits. They conclude that genome editing with a systems-level understanding would enhance abiotic stress tolerance [60,61,62].

The convergence of molecular biology, genomics, and biotechnology fast-track plant improvement, particularly under relatively simple Mendelian genetic architectures underlying abiotic stress responses [63,64]. However, these efforts, while useful at identifying and manipulating individual target functional variants [65], demand an understanding of how such molecular bases perform across more complex polygenetic and environmentally conditioned scenarios [66,67]. In other words, while functional validation and genome editing provide unprecedented precision to customize stress-responsive pathways [68], their ultimate impact will depend on integrating these advances within frameworks that explicitly account for genotype-by-environment interactions [69,70] and phenotypic plasticity [71], as discussed in the next section. In this sense, a catalog of functionally characterized and gene-edited alleles represents only one component to achieve resilience in plants, since the former must be contextualized within diverse environmentally conditioned responses [72,73,74,75] and broader phylogenetic backgrounds [76,77,78]. Bridging this gap requires moving beyond controlled model systems toward strategies that harness both bioengineered variation and naturally occurring adaptive diversity for pre-breeding [79] and conservation initiatives [80].

## 3. Plastic and Adaptive Responses for Climate-Resilient Breeding

The previous works exemplify how the evolutionary diversification across hidden layers of genetic redundancy and functional specialization can harbor adaptive potential to cope with environmental stressors, providing a repertoire of candidate genes for molecular breeding. Additionally, while advances in functional genomics and genome editing are rapidly improving our ability to identify and manipulate some of these variants, their ultimate value depends on how effectively these efforts can be translated into reliable and deployable plant populations under current environmental variability. In this context, phenotypic plasticity forecasting [17,81] and pre-breeding efforts to mobilize alleles from locally adapted germplasm to elite backgrounds [82] are required to handle the intrinsic genotype-by-environment interaction that more deterministic molecular models often overlook. Rather than viewing environmental-dependent responses as a limitation, emerging frameworks now recognize plastic clines as genetically controlled [83], selectable traits that can be harnessed to enhance resilience across heterogeneous and shifting climates [84]. At the same time, stress-adapted populations thriving in extreme or marginal environments provide access to cryptic allelic variation that is largely absent from elite breeding gene pools [85]. The following studies illustrate how integrating genotype-by-environment interactions and pre-breeding-oriented approaches can bridge the gap between functional discovery and application, ultimately enabling the development of plant populations and varieties able to cope with increasing abiotic pressures.

In an first effort to understand the genomic bases of phenotypic plasticity, Arenas and Cortés [86] re-studied drought-induced phenotypic plasticity in barley (*Hordeum vulgare*) using a diversity panel of 1277 genotypes evaluated under contrasting water regimes (i.e., well-watered vs. drought conditions). Using five plasticity indices and four genome-wide association study (GWAS) models, the authors identified 239 significant SNPs, including 27 within coding regions. Notably, many loci exhibited context-dependent antagonistic pleiotropy, where allelic effects differed across environments. Therefore, the study was able to conclude that plasticity is genetically controlled and environment-dependent, challenging traditional breeding strategies that assume stable allele effects. The work moves beyond single-locality mapping to explicitly model genotype-by-environment interactions, offering a conceptual and methodological framework for integrating phenotypic plasticity into genomic prediction and breeding strategies. Once these contrasting genetic responses are understood, they can be utilized to unlock hidden adaptive potential for crop improvement under increasingly variable water availability [87].

Meanwhile, the work by Yin et al. [88] advances the deployment of cryptic genetic diversity within a pre-breeding framework by demonstrating how salt-stress-adapted germplasm can be harnessed for crop improvement through molecular breeding. Focusing on rice, the authors identify and functionally validate two jacalin-related lectin genes—*OsJRL45* and *OsJRL40*—derived from the stress-resilient “Sea Rice 86” genotype, showing that their marker-assisted introgression significantly enhances salt tolerance. Because this study integrates gene expression analyses under salinity stress with breeding-oriented validation, it bridges the gap between gene discovery and downstream applications, highlighting lectin-mediated pathways as key components of stress response networks. Importantly, this work exemplifies how underutilized germplasm pre-adapted to or extreme environments harbors adaptive alleles absent from conventional breeding gene pools and how modern genomic tools can accelerate their deployment. In doing so, the study reinforces the importance of coupling functional genomics with marker-assisted selection to unlock hidden reservoirs of abiotic stress tolerance, providing a practical roadmap for developing resilient crop varieties in the face of increasing soil salinization.

Together, these two studies highlight the fact that climate-resilient breeding not only requires identifying exotic superior genotypes and alleles [89] and reconstructing the molecular mechanism responsible for that superiority [90], but also urge an understanding of how those networks are reshaped across environmental contexts [91,92] and genetic backgrounds [93,94]. Once phenotypic plasticity, genotype-by-environment interactions, and stress-adapted germplasm are explicitly incorporated into breeding pipelines, it would be possible to transcend beyond single-trait plant improvement strategies toward more predictive frameworks of the adaptative potential. These same principles spread beyond crops, as the identification and mobilization of context-dependent adaptive variation help sustain resilience in natural populations under rapid environmental change.

## 4. Leveraging Abiotic Stress Tolerance in Natural Populations

Moving beyond crop breeding applications, which tend to occupy a major focus in the abiotic stress tolerance literature, the same conceptual and methodological workflows used to study and harness cryptic adaptive variation and its environmental dependency can be extended to natural populations facing rapid environmental change. As climate pressures intensify, conservation [95], restoration [96], and assisted migration [97] and gene flow [98,99] efforts increasingly rely on identifying and mobilizing adaptive variation already present in wild and marginal plant populations. In this context, integrating genomics, transcriptomics, and *in situ* ecological data offers a powerful avenue to uncover how non-model and perennial species respond to abiotic stress across their range.

In a first effort in this line, Li et al. [100] examine cold tolerance in a mangrove species (*Kandelia obovata*) by comparing a reference cold-sensitive population and its transplanted counterpart to higher-latitude environments, revealing adaptive responses in a coastal woody plant under novel climatic conditions. The authors perform RNA-seq, differential expression analysis, SNP identification, and transcription factor prediction to identify DEGs under chilling stress. Their analyses reveal the enrichment of pathways related to phenylpropanoid biosynthesis, carbohydrate metabolism, and plant hormone signaling, alongside the differential regulation of key transcription factor families such as NAC, MYB, WRKY, and ERF [101,102,103,104], all of which are central to stress signaling networks. The work highlights that both genetic and post-transcriptional mechanisms underlie cold adaptation. Integrating experimental *in situ* population surveys with transcriptomic and functional analyses enables an understanding of how perennial species rely on regulatory strategies to expand their ecological niche, complementing the genomic basis of stress tolerance in non-model systems and reinforcing the importance of leveraging ecologically relevant germplasm for conservation and restoration under shifting temperature regimes.

Natural populations offer not only reservoirs of biodiversity, but also repositories of adaptive potential shaped by local environmental pressures [55]. Merging multi-omics screenings [105] with ecological and geographic metadata allows for the identification and mobilization of standing adaptive variation as part of conservation, restoration, and assisted adaptation strategies [106]. All compiled efforts (Table 1) deepen our understanding of the evolutionary genomic basis of local adaptation, while providing applied tools for conserving the genetic diversity that underlies abiotic stress tolerance, maintaining ecosystem resilience, and guiding targeted interventions under shifting climatic regimes.

## 5. Perspectives

**Figure 1:**
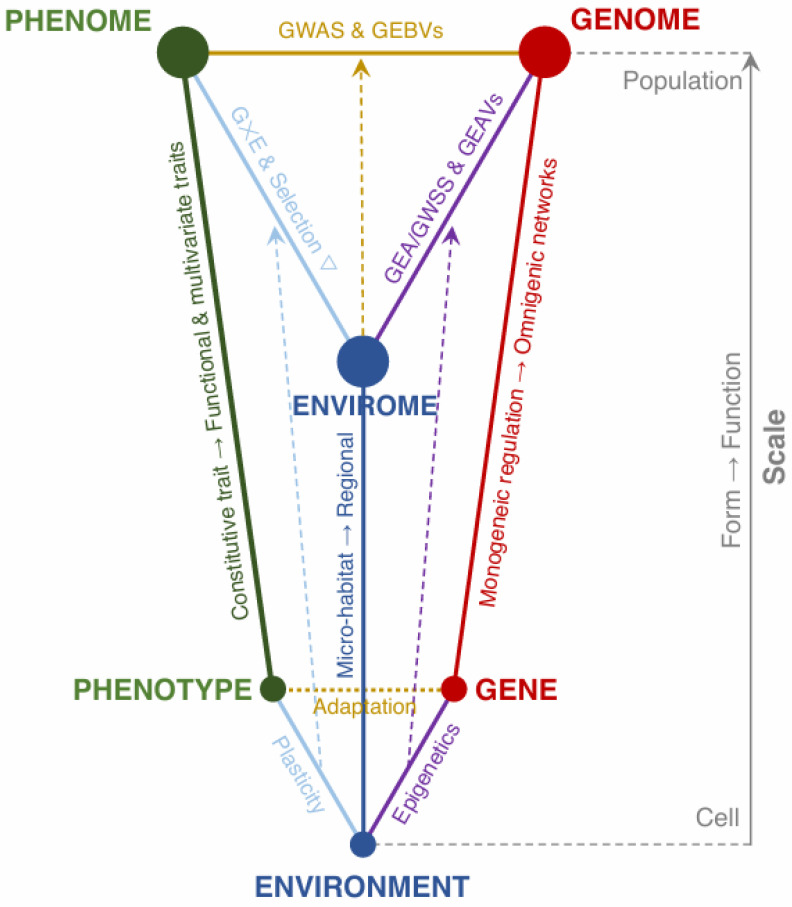
Conceptual framework depicting a tridimensional plant breeding triangle projected across spatial scales to leverage genetic diversity for abiotic stress tolerance under climate change. The diagram links the environment in which the plant experiences abiotic stress, the phenotypic variation that shapes tolerance, and the corresponding standing genetic variation enabling such plant responses. Together, these axes contextualize how environment–genotype–phenotype relationships interplay across spatial scales. The vertical dimension represents biological spatial organization, spanning from micro-habitats to regional climatic gradients for the environmental axis, from cellular monogenic processes to population-level polygenic dynamics for the genetic axis, and from a single constitutive trait to multivariate functional traits for the phenotypic axis. For instance, *(i)* the abstraction of the environment is no longer a discretization into few localities and climatic variables, but instead is currently acknowledging its continuum nature across space and environmental layers (i.e., envirome) through the enviromics paradigm. Similarly, *(ii)* at the molecular level, the transition from a monogenic understating to omnigenic regulatory networks implies that abiotic stress responses are now being recognized as governed by complex, interconnected gene systems rather than single major-effect loci, yet identifying some of the latter are still necessary for more targeted gene editing and biotechnological applications. The more complex poly/omnigenic level can jointly be attributed to coding variation, regulatory elements, and epigenetic modifications, as well as post-transcriptional processes such as alternative splicing, all of which account for the missing heritability of the more monogenic models. These molecular interactions collectively define the functional genome, which interacts with environmental stresses and the envirome to regulate a cascade of transcriptional and metabolic responses with implications on the tolerant phenotype. Finally, moving upward in scale, *(iii)* the phenomic axis depicts a continuum from constitutive traits to complex multivariate phenotypes that integrate developmental, physiological, and morphological functional responses. Such phenomic responses are shaped by phenotypic plasticity and genotype-by-environment (G × E) interactions, with the first mediating short-term acclimation through transgenerational epigenic inheritance and the second contributing to long-term adaptive potential, all of which serve as conceptual processes linking phenotype and environment (i.e., plasticity), phenotype and genotype (i.e., adaptation), and genotype and environment (i.e., epigenic responses). The framework also invites incorporating technical approaches and models borrowed from the plant breeding and eco-evolutionary disciplines capable of helping integrate all three axes. Specifically, analytical techniques such as genome-wide association studies (GWASs), genome–environment associations (GEAs), genome-wide selection scans (GWSSs), and genomic prediction and its corresponding genomic estimated breeding (GEBVs) and adaptive (GEAVs) values, enable the genomic architecture of stress tolerance (i.e., GWAS as a bridge between the phenotypic and genomic axes) and polygenic adaptation (i.e., GEA, GWSS and GEAVs as a link between the environmental and genomic axes) to be dissected and utilized to forecast climate change responses (i.e., GEBVs and GEAVs, respectively linking the phenotypic and environmental axes with the genetic axis). The cutting-edge integration of these approaches not only allow allelic variants and genomic regions associated with both functional trait performance and environmental adaptation to be identified, but also facilitate their deployment into pre-breeding and conservation programs. In parallel, the environmental filtering of functional phenotypes through natural and artificial selection can be assessed respectively using selection gradient and G × E analyses, unifying the phenomic and environmental dimensions. Last, the bidirectional feedback among phenotype, genotype, and environment reinforces the fact that adaptive responses to abiotic stresses are not solely a function of genetic determinants, but also of context-dependent expression and ecological interactions. This reflects the shift to a systems-level multi-dimensional understanding of abiotic stress tolerance, advocating for the integration of multi-omics data (e.g., genomics, transcriptomics, phenomics, and enviromics) with theorical developments from the ecological, evolutionary, and plant breeding fields. More importantly, by bridging scales (from genes to ecosystems) and processes (from molecular function to population adaptation), the roadmap offers a valuable guideline to harness cryptic genetic diversity for abiotic stress tolerance under rapid climate change in both crops and natural plant populations.

Taken together, the contributed studies on abiotic stress tolerance in plants reveal that the field is advancing rapidly in technical capacity but still grappling with conceptual, translational [107], and cross-disciplinary challenges [108,109]. A major persistent gap is the scale mismatch between molecular network discovery and ecological deployment. While transcriptomic experimental studies with moderate sample sizes provide high-resolution temporal snapshots of molecular stress responses, they capture only a fraction of the environmental variability experienced in the field. On the other hand, observational population-level analyses on hundreds and thousands of genotypes disclose complex genotype-by-environment interactions that are difficult to reconcile with epigenetic screening under controlled conditions. Bridging these scales together under the same lens would require experimental designs that integrate controlled setups with field-based validation [110] across environmental gradients [111,112] (Figure 1).

A second challenge lies in the fragmentation of multi-omics data. While individual studies employ transcriptomics, GWASs, or functional assays, few integrate these layers into cohesive predictive models. For example, candidate SNPs derived from GWAS models are not always directly linked to expression profiles or metabolic pathways, limiting their interpretability [113,114]. Similarly, so far, efforts to utilize transcriptomic signatures into predictive breeding frameworks are scarce. This dichotomy translates into a conceptual tension between reductionist and systems-level functional studies, which together would provide mechanistic clarity at the level of individual gene families and metabolic pathways, and population genomics assessments, capable of revealing highly polygenic environmental-dependent architectures. Reconciling these paradigms requires moving toward omnigenic reconstructions and network-based functional validation [115], where multiple genes and pathways are accounted for simultaneously. Merging multi-omics [116] and environmental data, across spatial, temporal and evolutionary scales [117,118], is now being assisted by machine learning and systems biology workflows [119,120,121,122].

Meanwhile, the compiled studies report a persistent underutilization of secondary gene pools, wild resources, and marginal populations, which are the ones more likely to harbor allelic variation useful for abiotic stress tolerance after thousands of years of co-evolution with local pressures. The richness of adaptive variation outside elite germplasm remains unattractable for most pre-breeding programs, not to mention conservation and reforestation efforts, most likely due to difficulties in assessing and accessing the adapted variants and divergent complex epistatic fitness architectures that preclude their direct trade-off-free use [123]. Therefore, approaches such as genomic-guided introgression and assisted gene flow must be scaled up to fully exploit these resources, together with translational societal efforts to increase adoption [107] and communitarian involvement [124].

Additionally, although genome editing has transformative potential, its scalation to complex traits remains limited. At the moment, while editing single genes is feasible, manipulating entire polygenic architectures is still a bottleneck. Future efforts must therefore focus on multiplex editing and regulatory network bioengineering [125], potentially targeting transcription factor hubs. Last, acknowledging that plasticity is not only environmentally modulated but also genetically controlled [126] is reshaping it as a breeding target, enabling plant improvement to utilize plastic and adaptive responses [87] as part of the nascent enviromics paradigm [127,128,129,130,131,132,133,134,135,136], enhancing resilience under variable climates.

## 6. Conclusions

This Special Issue reinforces the fact that abiotic stress tolerance is governed by complex regulatory networks across highly polygenic and environmentally dependent architectures, which cannot be fully captured through single-layer or reductionist approaches. Instead, boosting plant resilience would require integrating cryptic genetic variation, transcriptional regulation, alternative splicing patterns, and genotype-by-environment interaction across spatial and temporal scales. Moving forward, a key priority would be to integrate multi-omics datasets—including genomics, transcriptomics, phenomics, and environmental covariates—into more unified, predictive frameworks capable of linking causal variants to system-level phenotypes under heterogeneous, and even simultaneous, abiotic stresses. Achieving this integration will require advances in network inference and machine learning, and particularly joint experimental designs that explicitly couple controlled functional assays with population-level validation across environmental gradients. Scaling the utilization of exotic germplasm through genomic-enabled introgression, multi-target genetic editing, and assisted gene flow will help mobilizing standing adaptation from the wild. Abiotic stress resilience can no longer be harnessed through discrete gene targets, but requires rethinking it as interconnected omnigenic regulatory networks embedded within evolving populations and ecosystems.

## Figures and Tables

**Table 1:** Synthesis of the nine contributing studies to the Special Issue “Abiotic Stress Tolerance and Genetic Diversity in Plants” in its second edition. The table is ordered as per the narrative. Abbreviations: DEGs stand for differentially expressed genes, others are already declared within the table.

Species Group	Study Goal	Sampling	Omics Technologies	Key Findings	Reference
Cultivated alfalfa (*Medicago sativa*) & its wild relative (*Medicago falcata*)	Assess cold tolerance mechanisms in cultivated and wild seedlings	Expression screening 24 h and 120 h after low-temperature (4 °C)	RNA-seq	Wild *M. falcata* has broader transcriptional response with more DEGs	Wong et al. [33]
Soybean (*Glycine max*)	Validate three iron deficiency response genes (i.e., FIT, HY5 and PYE)	Iron-deficit-tolerant Swedish line Fiskeby III (PI 438471)	Virus-induced gene silencing and RNA-seq	Silencing FIT and HY5 does not compromise iron deficiency tolerance	O’Rourke and Graham [40]
Rice (*Oryza sativa*)	Dissect the molecular bases of salinity tolerance in rice	Salt-tolerant Thai variety ‘Jao Khao’	RNA-seq libraries sampled across six time points (0–48 h)	A total of 1950 variable and 111 hub genes linked with the salt tolerance	Khunsanit et al. [41]
Chinese kale (*Brassica oleracea* L. var. *alboglabra*)	Characterize abiotic stress response of *FCS-like zinc finger (FLZ)* genes	Chinese kale (*B. oleracea*), cabbage (*B. rapa*) and black mustard (*B. nigra*)	Phylogenomic, expression analyses, and protein–protein assays	FLZ proteins interact with central energy-sensing kinases (SnRK1)	Zhao et al. [42]
Rice (*Oryza sativa*)	Study variation in the sodium transporter *OsHKT1;1* for salt tolerance	Two splicing variants across three heterologous systems	Subcellular localization and expression analyses	Splicing variants differ in ion selectivity and transport efficiency	Imran et al. [56]
Alfalfa (*Medicago sativa*)	Review CRISPR/Cas in alfalfa for abiotic stress tolerance	Literature review	Genomics and transcriptomics	Systems-level genome editing benefits abiotic stress tolerance	Fan et al. [57]
Barley (*Hordeum vulgare*)	Reconstruct the genomic bases of plasticity to drought stress	Genotypes (1277) evaluated under well-watered vs. drought conditions	Five plasticity indices and four genome-wide association study (GWAS)	Drought stress plasticity is genetically and environment-dependent	Arenas and Cortés [86]
Rice (*Oryza sativa*)	Validate two genes (*OsJRL45* and *OsJRL40*) for salt stress tolerance	Salt stress-adapted “Sea Rice 86” and recombinant inbred lines (RILs)	RT-qPCR, yeast two-hybrid and bimolecular fluorescence	Marker-assisted introgression in RILs enhances salt tolerance	Yin et al. [88]
Mangrove (*Kandelia obovata*)	Examine cold tolerance in a cold-sensitive mangrove species	Cold-sensitive population transplanted to higher-latitude climates	RNA-seq	Genetic and post-transcriptional mechanisms underlie cold adaptation	Li et al. [100]

## Data Availability

All contributing articles to the Special Issue “Abiotic Stress Tolerance and Genetic Diversity in Plants”, second edition, are available here https://www.mdpi.com/si/ijms/9Y8E57B7V3 as accessed on 24 April 2026.
